# On-Orbit Signal-to-Noise Ratio Test Method for Night-Light Camera in Luojia 1-01 Satellite Based on Time-Sequence Imagery

**DOI:** 10.3390/s19194077

**Published:** 2019-09-20

**Authors:** Wei Wang, Xing Zhong, Zhiqiang Su

**Affiliations:** 1 Changchun Institute of Optics, Fine Mechanics and Physics, Chinese Academy of Sciences, Changchun 130033, China; wangwei815@mails.ucas.ac.cn; 2 College of Materials Sciences and Opto-Electronic Technology, University of Chinese Academy of Sciences, Beijing 100049, China; 3 Chang Guang Satellite Technology Co., LTD, Changchun 130102, China; suzhiqiang@charmingglobe.com

**Keywords:** SNR, night-light, time-sequence imagery, Luojia 1-01 satellite, low-resolution, on-orbit test, radiometric calibration

## Abstract

Night-light remote sensing imaging technologies have increasingly attracted attention with the development and application of focal plane arrays. On-orbit signal-to-noise ratio (SNR) test is an important link to evaluate night-light camera’s radiometric performance and the premise for quantitative application of remote sensing imageries. Under night-light illumination conditions, the illuminance of ground objects is very low and varies dramatically, the spatial uniformity of each pixel’s output cannot be guaranteed, and thus the traditional on-orbit test methods represented by variance method are unsuitable for low-resolution night-light cameras. To solve this problem, we proposed an effective on-orbit SNR test method based on consecutive time-sequence images that including the same objects. We analyzed the radiative transfer process between night-light camera and objects, and established a theoretical SNR model based on analysis of the generation and main sources of signal electrons and noise electrons. Finally, we took Luojia 1-01 satellite, the world’s first professional night-light remote sensing satellite, as reference and calculated the theoretical SNR and actual on-orbit SNR using consecutive images captured by Luojia 1-01 satellite. The actual results show the similar characteristics as theoretical results, and are higher than the theoretical results within the reasonable error tolerance, which fully guarantee the detection ability of night-light camera and verify the validity of this time-sequence-based method.

## 1. Introduction

With the development of focal plane detectors and the needs of engineering applications, night-light remote sensing imaging technologies have increasingly attracted more attentions from remote sensing, economics and other research fields [1]. As early as the 1960s, the US defense meteorological satellite carried a low-light-level imaging camera to carry out meteorological observation for strategic reconnaissance [2,3,4]. Night-light remote sensing cameras make up for the shortcoming that most cameras can only observe in daytime, which enhance the timeliness of information acquisition.

Night-light remote sensing imaging is of great importance in engineering applications. By long-term observation of the same ground objects under low illuminance, we can extract information such as vehicles [5] and draw night-light maps [3,6]. Taking night-light images as the medium of economic analysis, we can evaluate the socio-economic situation such as urbanization process [7,8,9,10,11] and gross domestic products [12] of different regions. Besides, night-light images can reflect the law of human activities and the impact on local regions, such as light pollution [13,14,15] caused by abuse of artificial light sources [16], water storage [17], urban heat island effect [18,19], housing vacancy rate [20], and population density [21], even the impact on disease spread [22] and the state of war in hot spot [23].

Analysis of SNR (signal-to-noise ratio) combined with radiometric calibration is the basis and premise for quantitative applications of night-light images including the applications mentioned above. SNR is an important technical index to evaluate image quality and radiometric characteristics of cameras, which can be used to describe the ability of cameras to distinguish objects under different radiance [24,25]. SNR determines the amount of information that images could provide, and ultimately influences the accuracy of detecting and recognizing. The higher the SNR, the stronger the anti-noise interference ability of imaging system.

The image quality of night-light cameras degrades rapidly with the attenuation of radiant flux received by cameras. When the signal electrons number degenerates to the same order of magnitude as noise electrons number, the cameras can hardly detect any object. Therefore, theoretical analysis and on-orbit test of SNR is of vital importance to night-light cameras. The variance method and its derivative methods are wildly used among the existing on-orbit SNR test methods currently [26]. As long as the outputs of each pixel from the selected large regions of image detector are consistent or uniform, we can get a reliable SNR result. However, under night-light illumination conditions, the illuminance of ground objects is very low in general, and the illuminance changes dramatically due to the change of lighting sources [27]. For low-resolution cameras, it is difficult to guarantee the invariant condition of pixels’ output, which means the variance method is no longer applicable. It is urgent to find an effective on-orbit SNR test method for low-resolution night-light cameras.

Luojia 1-01 satellite is the world’s first professional night-light remote sensing satellite, mainly used for global night-light remote sensing and validation the enhancement technology of satellite navigation, providing data for macroeconomic analysis based on night-light remote sensing [25,27]. Luojia 1-01 satellite, developed by Chang Guang Satellite Technology Co., Ltd and Wuhan University, which have been successfully launched on 2 June 2018, could provide night-light remote sensing images with 129 m spatial resolution and 15 bits dynamic range. The main optical load of the Luojia 1-01 satellite is night-light remote sensing camera with high sensitivity imaging detector and large relative aperture optical system, which can draw the global nighttime light image within 15 days.

Aiming at the problems above, this paper proposed an on-orbit SNR test method for night-light cameras based on time-sequence images, which is quite different from the usual used methods based on spatial-sequence images represented by variance method. This method provides an effective SNR calculation method aiming at low-resolution night-light cameras and less uniform images, breaks through the limitation of resolution to the traditional SNR calculation method such as variance method. Firstly, we analyzed the transfer process of radiation between ground objects and night-light cameras in detail, and established a full-link radiative transfer model of night-light remote sensing. Then we analyzed the generation and main sources of signal electrons and noise electrons based on the radiative transfer model, and we further established the theoretical SNR model of night-light cameras in form of signal electrons and noise electrons. Finally, to verify the validity of this method, we took the night-light camera carried by the Luojia 1-01 satellite as example and calculated the theoretical SNR using its system parameters.

## 2. On-Orbit SNR Test Method

On-orbit SNR test is the premise of quantitative application of remote sensing images, that is, using images captured by remote sensing camera to calculate the actual SNR when the satellite works on orbit, and finally establish the corresponding relationship between SNR and radiation of ground objects. Radiometric calibration is used to establish the relation between the output of detector and the radiance entering the entrance pupil, determines the accuracy of radiometric calibration [24,25].

### 2.1. Spatial-Sequence-Based SNR Test Method

Various on-orbit SNR test methods have been proposed so far [26,28,29,30,31], which include the variance method [32], correlation method [33], artificial neural network [34], and other less commonly used methods such as the information entropy [35] method and spatial structure method [36]. The variance method and local variance method are based on single image, the former requires large and uniform area on the image, while the latter divides less uniform image into smaller part for further processing. However, these two methods have high requirement for resolution. It is hard to find a uniform area in low-resolution image, reduces the accuracy of these methods. The correlation method is mainly used for hyper-spectral images, which removes the signal by linear regression so as to separate the noise. An artificial neural network imitates the behavioral characteristics of animal neural network and can be used for measuring any image, but this method requires a large number of multi-state samples. Information entropy and spatial structure method evaluate image quality from new perspectives, but they are very rare in practical applications. Among these methods, the variance method and its derivative methods such as local variance method [37,38] have become the most wildly accepted methods due to the fast operation speed and wide applicability. Taking the variance method as reference, when using the variance method, first of all, we need to select a flat region with large area and uniform brightness distribution from single remote sensing image. Then calculating the average value and standard deviation of all the pixels’ output in the selected region. Generally, SNR is defined as the ratio of average value and standard deviation, i.e.,

$$\left\{\begin{array}{l} S_{e}=\bar{x}=\left[\sum x_{i}\right]/n\\ N_{e}=\sqrt{\left[\sum {\left(x_{i}-\bar{x}\right)}^{2}\right]/\left[n-1\right]}\\ SNR=S_{e}/N_{e} \end{array}\right.$$

where *n* is the total number of pixels in the sampling region, *x_i_* is the output value of each pixel, *S_e_* and *N_e_* are average value and standard deviation of the selected region respectively. As long as the spatial consistency of each pixel’s output can be guaranteed, we can utilize these methods to acquire reliable results.

### 2.2. Limitation of Night-Light Remote Sensing

Under low-light-level or night-light illumination conditions, when capturing images for ground objects with low illuminance, in order to obtain higher SNR, it would inevitably lead to the loss of the camera’s spatial resolution. Table 1 listed the spatial resolution of the existing optical remote sensing satellites that can acquire images under night-light illumination conditions.

**Figure 1:**
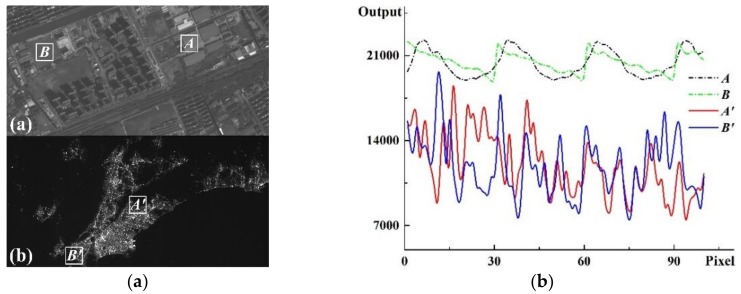
Comparison of (**a**) high-resolution image and low-resolution image and (**b**) the output values curves of 4 different regions from the two images.

It can be seen from Table 1 that almost all the existing optical remote sensing satellites have a low resolution, thus the application of variance method faces great difficulties here. Taking the night-light camera carried by the Luojia 1-01 satellite as example [27], on the orbit with an altitude of 645 km, the spatial resolution is about 129 m, which means that a ground object with an area of 129 × 129 m^2^ is projected onto a single pixel with an area of 11 × 11 μm^2^. Under such low-resolution, all the objects in the camera’s field of view are severely discretized, and the detail characteristics of the objects are almost all ignored. Under night-light illumination condition, the illuminance of objects is severely restricted by lighting sources, which lead to the dramatic change in the distribution of illuminance. We can hardly find a region with uniform brightness distribution from these low-resolution images to calculate SNR, thus the variance method is no longer applicable. Figure 1a shown the images respectively captured by high-resolution camera of the Jilin-1 satellite and low-resolution camera of the Luojia 1-01 satellite. We could see the playground and roof clearly in high-resolution image, but we just saw the outline of city in low-resolution image. We selected 4 regions from these images as much as possible to satisfy the requirement of variance method, and the output of each pixel is shown in Figure 1b. It is shown in Figure 1b that the standard deviation of region *A* and *B* are 1120.9 and 871.0, while those of region *A′* and *B′* are 2910.3 and 2856.8. The outputs of high-resolution image are more uniformly distributed than those of low-resolution image. Therefore, the variance method is no longer applicable to the on-orbit SNR test for low-resolution night-light cameras.

### 2.3. Time-Sequence-Based SNR Test Method

Though only one image is used, the variance method is essentially a method based on spatial sequence images, because the selected region with uniform brightness distribution forms a sequence of pixels in space. Assuming that the total number of pixels in selected region of image detector is *N*, the uniform brightness distribution in this region means that the output values of these *N* pixels in this spatial sequence is approximately equal. If we take these pixels as imaging process one after another, it is equivalent to acquiring *N* images of ground objects with similar or identical radiometric characteristics. Further, we could consider it as continuous photograph of the same ground object in a certain time-sequence. Based on this idea, we can convert the spatial-sequence-based method into time-sequence-based method. For the satellite operates in push-broom mode, it could acquire a series of images consecutively when executing an imaging tasks at nighttime. For instance, the working time of the Luojia 1-01 satellite is about 60 s during an imaging task, and the exposure time of the camera is about 13.7 ms. Except the extra time required by other operations such as charge transfer and electronic processing, the camera can acquire dozens of images theoretically. Then we can extract the images with the same ground object and compose these selected images into time-sequence images.

**Figure 2:**
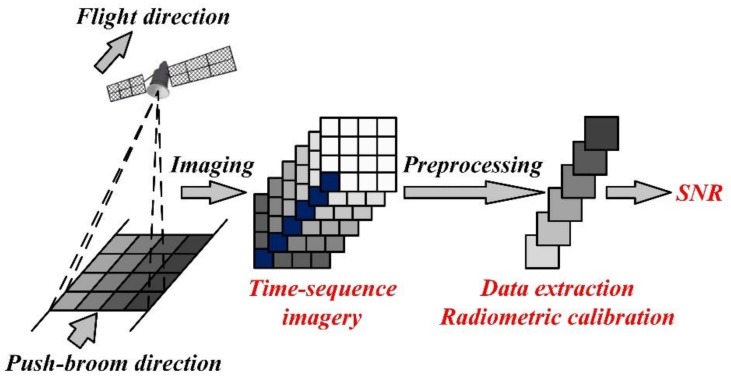
On-orbit signal-to-noise ratio (SNR) test process based on time-sequence images.

The process of this new SNR test method is shown in Figure 2. Firstly, the continuous night-light images captured during a continuous time that including the same ground object are selected. In order to improve the reliability, at least 10 consecutive images are selected. There is no doubt that these images cannot be applied directly, thus these images need to be pre-processed such as registration and the saturated pixels caused by excessive irradiance need to be eliminated before applying. Then, the data of the same ground object is extract from these processed images and arranged into a data sequence. Finally, the average value and standard deviation of these extracted data are calculated as the variance method did. Combined with the radiometric calibration data, we could obtain the on-orbit SNR under specific spectral irradiance or illuminance.

During the consecutive imaging process, the relative positional relationship between objects and camera changes constantly. Therefore, the proposed method in this paper requires the bidirectional distribution characteristics of objects to be isotropic [39]. Most of the ground objects can be regarded as Lambert radiators [40], and the bidirectional reflectance distribution can be neglected. However, for objects with anisotropic scattering such as water, the use of this method is still questionable.

## 3. Radiative Transfer Model

**Figure 3:**
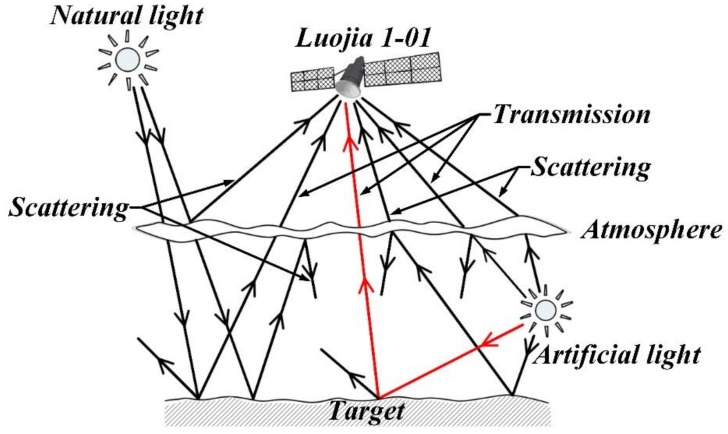
Radiative transfer process of night-light remote sensing.

Under night-light illumination conditions, the radiative transfer process between ground objects and space camera is shown in Figure 3 [41,42]. The light emitted from lighting source is transmitted to the surfaces of ground objects, and then transmitted into atmosphere after reflected off the surfaces. Some of the light from ground objects transmitted into the camera’s entrance pupil after atmospheric transmission and atmospheric scattering. Meanwhile, some of the light from lighting sources is also transmitted into the camera through atmospheric transmission and atmospheric scattering. Then the optical system converges the light distributed in the operation waveband to the focal surface (the surface of image detector) and then the image detector converts these collected photons into electrons with a certain conversion efficiency according to the photoelectric conversion principle. The electrons were stored as initial data after circuit amplification and analog-to-digital conversion.

Lighting sources include natural light sources and artificial light sources [43]. The natural light mainly refers to light reflected by the moon or other stars or light emitted by these stars themselves. While the artificial light generally refers to the lamplight, whose radiation in most cases is transmitted downward, and only in rare cases it can be directly radiated to upper atmosphere. Different lighting sources have different brightness or illuminance [44,45]. Although the moon and other stars have very high brightness, the distance between natural light sources and ground objects is so long that the illuminance relative to the ground is quite small [14]. The illuminance of the moon relative to the ground is about 2.67 × 10^−1^ lx, even for the brightest Sirius, the illuminance is only about 1.39 × 10^−4^ lx due to the long distance from Sirius to earth [40,46]. The distance between artificial light and ground objects is far less than that of natural light, so the illuminance relative to the ground is far greater than that of natural light, which is usually distributed in the range of 10^1^~10^3^ lx [40,46]. Therefore, only artificial light is considered in radiative transfer model of night-light remote sensing [44,47].

**Figure 4:**
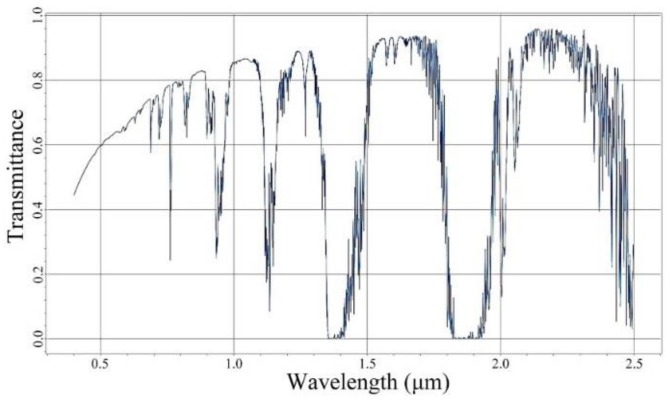
Atmospheric transmittance curve (atmospheric windows).

Atmospheric transmission is the most important way of light propagation in remote sensing. Though atmospheric transmittance varies with the change of wavelength, the transmittance during atmospheric windows is generally maintained above 60%. Under condition of cloudless or rainless, and rural aerosol model with visibility of 23 km, the atmospheric transmittance of each wavelength calculated by MODTRAN is shown in Figure 4 [48].

Scattering here refers to the atmospheric scattering [49,50], mainly including Rayleigh scattering and Mie scattering, and the intensity of Mie scattering is similar to that of Rayleigh scattering. Taking Rayleigh scattering as example, under the standard atmospheric pressure, the Rayleigh scattering coefficients at the wavelength of (680, 550, 440) nm are about (5.8, 13.5, 33.1) × 10^−3^ km^−1^, and the scattering transmittances of these three wavelengths at the height of 645 km are (2.37 × 10^−2^, 1.65 × 10^−4^, 5.346 × 10^−10^), much smaller than atmospheric transmittance [51,52]. Even for the long wave band that insensitive to scattering, the scattering transmittance decreases in geometric series after multiple atmospheric scattering, thus the light returning to camera’s entrance pupil can be neglected.

Based on the above analysis, the radiative transfer model of night-light remote sensing focus on the radiation that enters camera’s entrance pupil after atmospheric transmission, the radiation mainly consists of artificial light reflected by ground objects, as the part marked with red lines in Figure 3.

## 4. Theoretical SNR Model

SNR is usually defined as the ratio of signal electrons number to noise electrons number, but it is usually expressed in form of decibel (dB) in engineering applications as follow

(1) $$SNR=20\log_{10}\left(\frac{N_{signal}}{N_{noise}}\right).$$

### 4.1. Signal Electrons Model

According to the radiative transfer model, we can deduce and establish the theoretical SNR model in form of photon. The signal electrons generated by a single pixel is related to the reflection characteristics of ground objects, physical characteristics of detector, optical system, atmospheric transmittance, quantum efficiency, and other factors. Tracking the radiation entering camera’s entrance pupil is a more convenient method to analyze the generation of signal electrons.

**Figure 5:**
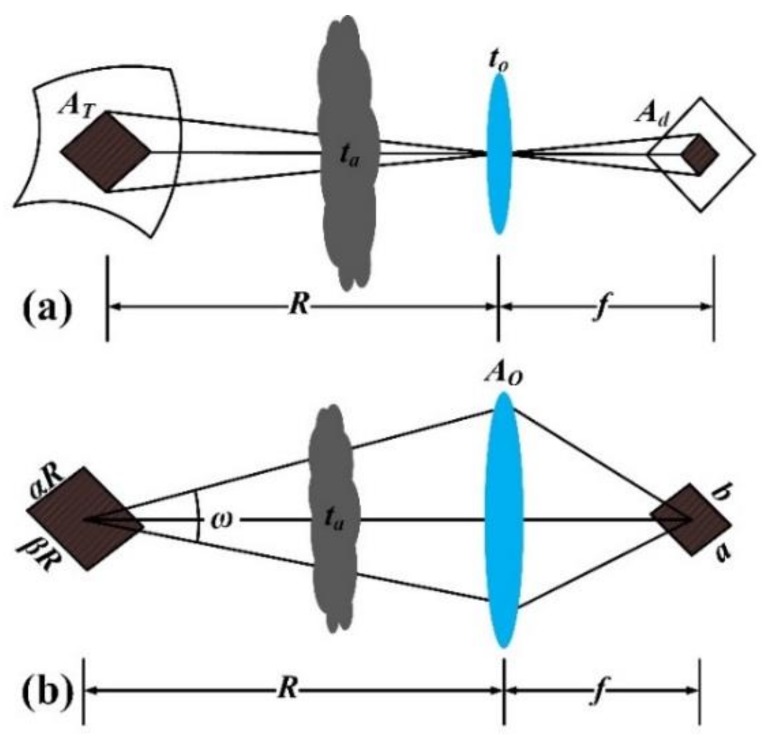
(**a**) Imaging process and (**b**) radiative transfer process between certain ground object and single pixel of image detector.

The relationship between ground object and single pixel in remote sensing imaging is shown in Figure 5. Figure 5a shows the process that certain ground object projected onto a single pixel. Figure 5b shows the process that within a certain spatial solid angle, the radiation emitted by ground object enters optical system after atmospheric transmission and finally acquired by detector.

In Figure 5, *t_a_* and *t_o_* are atmosphere transmittance and optical transmittance, *R* and *f* are satellite’s orbit altitude and night-light camera’s focal length, and *A_o_* is the area of camera’s entrance pupil. *A_d_* is the area of single pixel, *a* and *b* are the size of single pixel, which satisfy *A_d_ = a* × *b*. *A_T_* is the area of single pixel, *α* and *β* are solid angles of ground object relative to camera’s entrance pupil, which satisfy *A_T_ = αR* × *βR*. According to the geometric similarity principle, the values of *α* and *β* are the same as the solid angle of single pixel relative to camera’s pupil, i.e., *αβ = ab/f*^2^. *ω* is the solid angle of camera’s entrance pupil with respect to ground object, which satisfy *ω* = *A_o_*/*R*^2^.

In the spectrum of *λ~λ +* ∆*λ*, assuming that the ground object in Figure 5 is an extended radiation source. Assuming that the angle between normal line and radiation center of the extended radiation source satisfying *θ* = 0, the solid angle of the camera’s entrance pupil relative to the extended radiation source is Ω. Assuming the quantum efficiency of detector is *η*. In quantum theory, the energy carried by single photon is *hν*. For a camera without occlusion, if the radiant flux *Q_e_* collected by one single pixel is divided by *hν*, we can obtain the signal electrons number *N_signal_* in exposure time *T*_int_.

(2) $$N_{signal}=\frac{Q_{e}}{h\nu }=\frac{L_{e}(\lambda )t_{a}(\lambda )t_{o}(\lambda )A_{T}\Omega \eta (\lambda )T_{\mathrm{int}}\cos\theta }{h\nu }=\frac{\pi A_{d}L_{e}(\lambda )t_{a}(\lambda )t_{o}(\lambda )\eta (\lambda )T_{\mathrm{int}}}{4F^{2}h\nu },$$

where the area of entrance pupil is replaced by diameter, and *F* is relative aperture of optical system. By integrating the wavelength within the operation waveband, we obtain the following formula of signal electrons number in integral form

(3) $$N_{signal}=\frac{\pi A_{d}T_{\mathrm{int}}}{4F^{2}h\nu }\int_{\lambda }L_{e}(\lambda )t_{a}(\lambda )t_{o}(\lambda )\eta (\lambda )d\lambda .$$

In Equation (3), the characteristics of extended radiation source, atmospheric transmittance, and quantum efficiency are all functions of wavelength. We can obtain the specific form of these parameters relating to wavelength, however that is difficult for night-light remote sensing imaging. To solve this problem, we use the average values in operation waveband to replace these wavelength-related parameters approximately, so Equation (3) is eventually modified as0

(4) $$N_{signal}=\frac{\pi A_{d}T_{\mathrm{int}}}{4F^{2}h\nu }L_{e}t_{a}t_{o}\eta ,$$

where *L_e_* represents the integral radiance of ground objects that entering camera’s entrance pupil. Compared with Equation (4), the parameters without *λ* as subscript represent the average values of the corresponding parameters that relating to wavelength.

### 4.2. Noise Electrons Model

**Figure 6:**
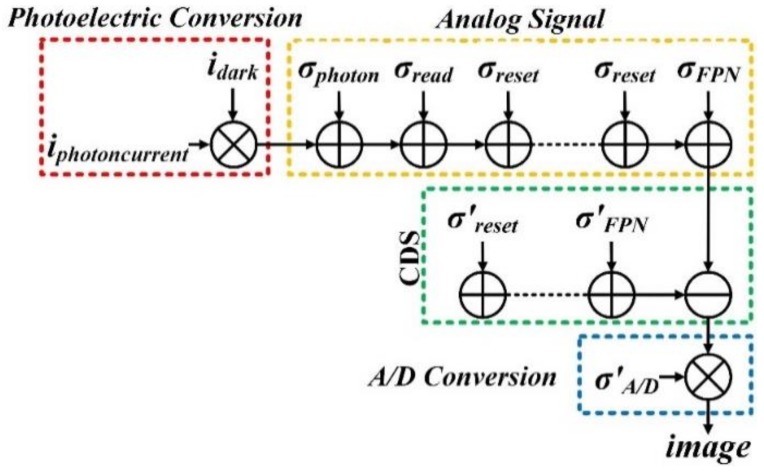
Charge transfer process and noise sources of image detector.

Noise is the superposition of many factors that interfere with signal by certain mathematical operation, which is mainly generated in charge transfer process. The charge transfer process shown in Figure 6 including photoelectric conversion process, analog signal generation process, correlated double sampling process (CDS) and analog-to-digital conversion process (ADC) [53,54]. It is shown that during the exposure time, the photocurrent and dark current generate noise in the process of generating analog signal, the fixed pattern noise and reset noise can be suppressed or eliminated by radiometric calibration and CDS technology, finally the analog signal can be converted into digital signal. Noise sources are very complex, mainly including photon shot noise, dart current noise, fixed pattern noise, circuit readout noise, reset noise, and quantization noise [55,56]. Therefore, only the photon shot noise, dark current noise, readout noise and quantization noise are considered.

• Photon shot noise

Photon shot noise is inherent properties of detector, related to the random incident of photons and cannot be reduced. The random incident of photons results in the random generation of photoelectrons. The SNR increases with the increase of shot noise. The standard deviation of photon shot noise is approximately equal to the square root of signal electrons number $\sigma_{photon}=\sqrt{N_{signal}}$.

• Dark current noise

Due to the thermal excitation effect of semiconductor, even in dark condition, detectors with certain temperature can generate current due to the irregular thermal notion of electrons, thus dark current noise occurred. The standard deviation of dark current noise approximately equals the square root of dark current electrons number $\sigma_{dark}=\sqrt{D_{e}}$.

• Circuit readout noise

Circuit readout noise mainly refers to amplifier noise of readout circuit, which is closely related to the circuit structure of detector. Readout noise is additive noise, no matter how many signal incidents to one single pixel, the noise always accumulated to signal separately. The circuit readout noise can be obtained from user’s manual.

• Quantization noise

In the ADC process, analog signal is quantified to be 2*^b^*-level digital signal, which has significant impact on image quality. Quantization noise is defined as the difference of signal before and after quantization, related to reference voltage, quantization bits, and system gain. The standard deviation of quantization noise is usually expressed as$\;\sigma_{A/D}=N_{FW}/\left(2^{b}\sqrt{12}\right)$, here *N_FW_* is the full well capacity.

Although the sources of noise are very complex, each component of noise is independent and non-correlated, their influences on SNR are equivalent. According to the principle of independent error synthesis, the total noise can be expressed as the square root of the sum of squares of each noise component, then the noise electrons number of night-light camera can finally be expressed as

(5) $$N_{noise}=\sqrt{\sigma_{photon}^{2}+\sigma_{dark}^{2}+\sigma_{read}^{2}+\sigma_{A/D}^{2}}=\sqrt{N_{signal}+D_{e}+\sigma_{read}^{2}+\frac{N_{FW}^{2}}{2^{2b}\times 12}}.$$

### 4.3. Conversion of Radiometry and Photometry

The intuitive data we can directly obtain in actual measurement is photometry, so it is necessary to convert these formulas above derived based on radiometry into photometry [57]. According to the definition, illuminance is used to represent the luminous flux received by surface per unit, and luminosity is used to represent the luminous flux emitted by unit surface. Considering reflection only, the same ground object is both receiving surface and emitting surface, whose illuminance and luminosity are the same in value [40], i.e., *M_ν_* = *E_ν_*. The luminosity cannot represent the light-emitting characteristics of lighting sources in different directions, so the luminance is needed to be a medium for calculation. For most of the ground objects that can be regarded as Lambert radiators with cosine radiometric characteristics, the relationship between luminosity and luminance satisfying *M_v_* = *πL_v_*.

Radiometry and photometry are different descriptions of the same phenomenon [58], the media that connects these two physical quantities is spectrum power function [59]. Though spectrum power function varies with the change of wavelength, it is usually taken as 680 lm/W, i.e., the radiant energy of 1 W at the most sensitive wavelength of visible light is about 680 lm in a space with a solid angle of 4π formed by uniform point lighting source. In the space with a solid angle of 2π, assuming that the reflectance of ground object is *ρ*, then the radiance and luminance satisfying the following relation

(6) $$L_{e}=\frac{2}{680}\cdot L_{v}=\frac{2}{680}\cdot \frac{E_{v}\rho }{\pi }.$$

Taking the formulas above into Equation (1), the theoretical SNR model is finally expressed as

(7) $$SNR=20\log_{10}\left(\frac{\frac{\pi A_{d}T_{\mathrm{int}}}{4F^{2}h\nu }\cdot \frac{E_{v}\rho t_{a}}{340\pi }\cdot t_{o}\cdot \eta }{\sqrt{\frac{\pi A_{d}T_{\mathrm{int}}}{4F^{2}h\nu }\cdot \frac{E_{v}\rho t_{a}}{340\pi }\cdot t_{o}\cdot \eta +D_{e}+\sigma_{read}^{2}+\frac{N_{FW}^{2}}{2^{2b}\times 12}}}\right).$$

## 5. Results and Discussion

### 5.1. Theoretical Prediction of SNR

Table 2 and Table 3 listed the parameters of the Luojia 1-01 satellite and image detector respectively.

**Figure 7:**
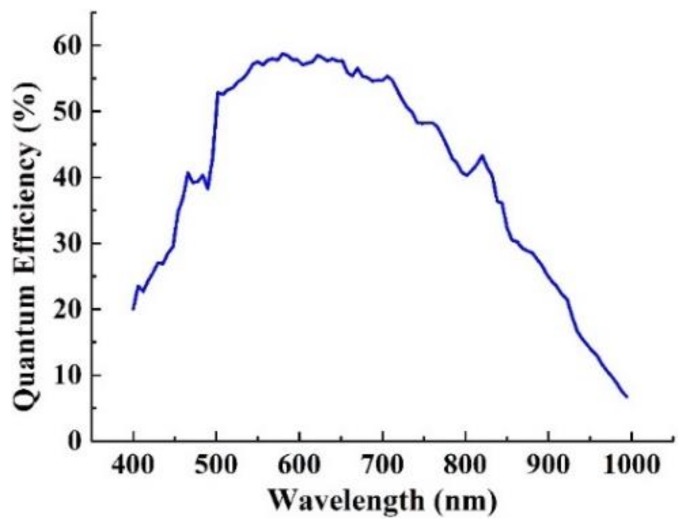
Quantum efficiency curve of image detector.

In the waveband of 0.5~0.9 μm, the average atmospheric transmittance *t_a_* calculated from Figure 4 is about 0.682. Under night-light illumination condition, the typical reflectance of ground objects is about 0.3. The quantum efficiency is the proportion of electrons number produced by detector to the photons number that incident to detector in exposure time. The quantum efficiency curve is shown in Figure 7, and the average quantum efficiency *η* in the spectrum of 0.5~0.9 μm is about 0.52.

Under the push-broom mode, the image of object projected on detector varies with the motion of satellite. In order to guarantee the image quality, the exposure time is limited by velocity-to-height ratio, i.e., *T_int_* ≤ *GSD*/*V_R_*, which means the motion of image on detector is less than one pixel, the ground velocity of satellite *V_R_* can be calculated according to Newton’s law of gravitation. On the orbit with altitude of 645 km, the exposure time satisfying *T_int_* ≤ 18.86 ms.

**Figure 8:**
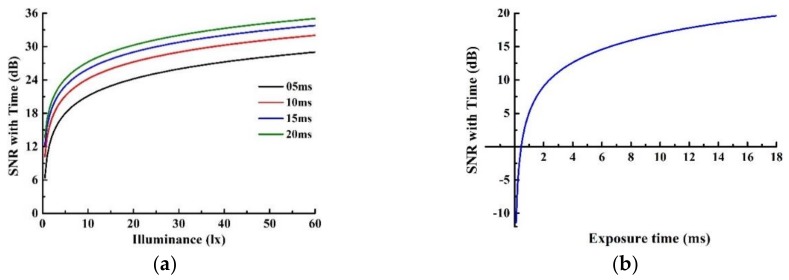
Curves of SNR versus (**a**) illuminance under different exposure times and (**b**) exposure times under certain illuminance.

According to the above parameter settings, the theoretical SNR curve of the night-light camera is shown in Figure 8. Figure 8a shows the SNR curves that vary with illuminance under different exposure times. As the exposure time increase from 5 ms to 20 ms, the SNR improved as a whole, but the increase is getting slower. Figure 8b shows the change of SNR curve with exposure time under certain illuminance, where the illuminance is fixed to be 2 lx. With the decrease of exposure time, the negative SNR appeared, i.e., the signal electrons number is less than noise electrons number, which means the image cannot be used.

### 5.2. On-Orbit Test of SNR

With the purpose of calculating the actual on-orbit SNR of night-light camera, we collected a series of consecutive time-sequence night-light images from Mexico City, New Delhi and Columbia captured by the Luojia 1-01 satellite during consecutive imaging time. After pre-processing such as image registration, we extracted multiple consecutive image points of different ground objects from these three time-sequence-image series, and then analyzed the data statistically according to the proposed method introduced in Section 2.3. Table 4 lists the working status and parameters setting of image detector when taking images for these three regions.

Radiometric calibration uses integrating sphere as lighting sources and the light outputted from integrating sphere is collected by camera. Changing the light intensity of integrating sphere and monitoring it with a spectral radiometer, we can obtain the radiometric calibration efficiency by statistical analysis of the images captured during calibration process. According to radiometric calibration results, taking the radiance and output of detector as *x*-axis and *y*-axis respectively, there is a linear relation between radiance entering camera’s entrance pupil and output of image detector. Under HDR recording mode, as the exposure time changes, the radiometric calibration coefficients under the gains of 1.85x and 3.68x are shown in Table 5, the dimension of slope is (W/m^2^/sr)^−1^. Using the data in Table 5, we can fit the radiometric calibration coefficients under exposure time of 13.7 ms. The linear responses of 1.85x in HDR low-gain mode and high-gain mode are *DN* = 11974.35 × *L_e_* + 211.59 and *DN* = 114697.89 × *L_e_* + 168.77 respectively, while those of 3.68x in HDR low-gain mode and high-gain mode are *DN* = 23893.58 × *L_e_* + 208.14 and *DN* = 243341.60 × *L_e_* + 143.07.

According to the radiometric calibration data, excluding the influence of saturated points and dark fields, we calculated the average value and standard deviation of sampling points extracted from time-sequence images of these three selected regions respectively. The statistical results are shown in Table 6, which illustrates that within HDR high-gain mode, the output values and the distribution of actual on-orbit SNR of the sampling points have similar characteristics.

**Figure 9:**
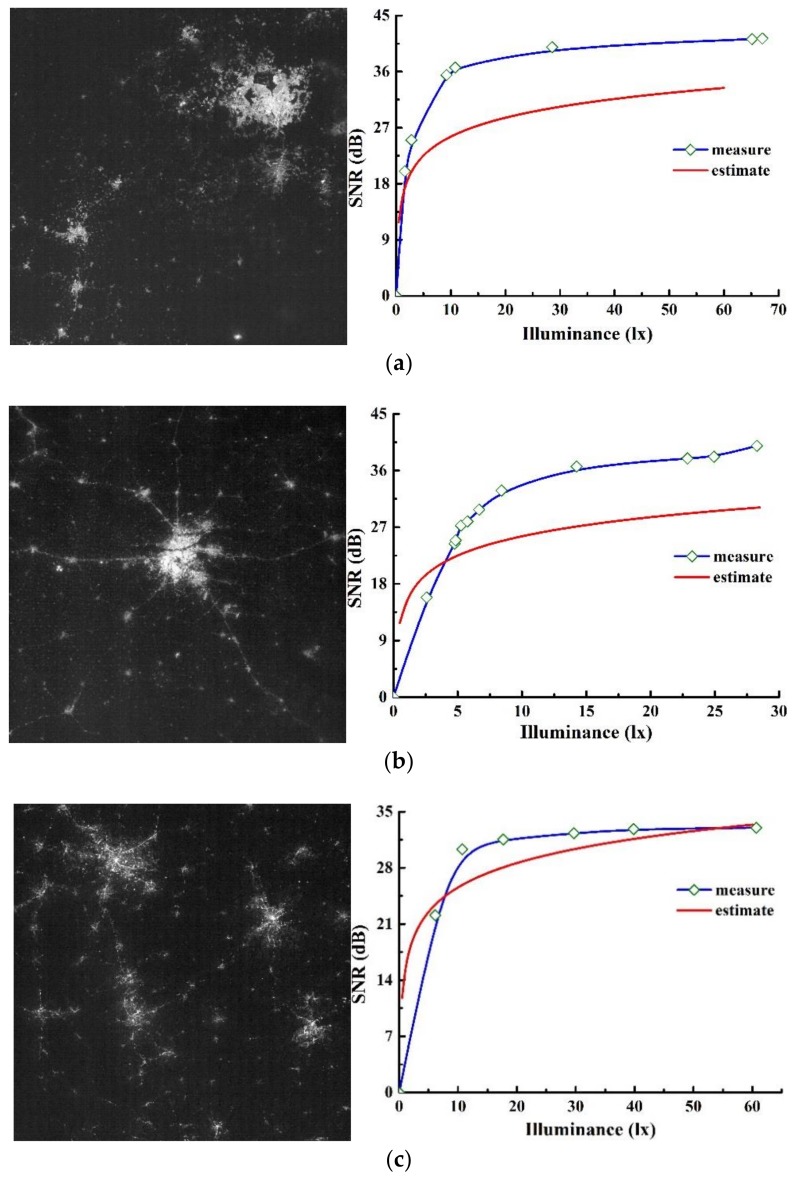
Night-light images and on-orbit test results of SNR based on time-sequence imagery of (**a**) Mexico City, (**b**) New Delhi, and (**c**) Columbia.

The night-light images and the specific on-orbit SNR test results of these three regions when the detector works on HDR high-gain mode are shown in Figure 9. The left column shown these three regions, and the two-dimensional curves are SNR curves that vary with illuminance, in which the blue curves are actual on-orbit SNR test results obtained by the time-sequence-based method, the red curves are theoretical SNR prediction results. It can be seen from these figures that actual SNR results have the same variation tendency and data distribution characteristics as the theoretical SNR results. Typical, under the illuminance of 10 lx at night, only when the SNR of typical objects at least reaches 20 dB can it satisfy the requirement of engineering applications. The curves in indicate that the theoretical SNR is about 25.6 dB under 10 lx illuminance, while the on-orbit SNR are about respectively 36.1 dB, 34.4 dB, and 29.0 dB, higher than application requirement and theoretical value.

It is shown in Figure 9 that when the illuminance is lower than a certain threshold (this threshold varies in different scenes, that is about 10 lx in Columbia and about 5 lx in Mexico City and New Delhi), SNR increases rapidly with the increase of illuminance. However, the theoretical prediction SNR are slightly higher than the actual measured SNR. One of the reasons for these differences is that although the Luojia 1-01 satellite has been equipped with the wide dynamic range and high sensitivity detector, the imaging capability is still limited by the performance of detector under ultra-low illuminance.

**Figure 10:**
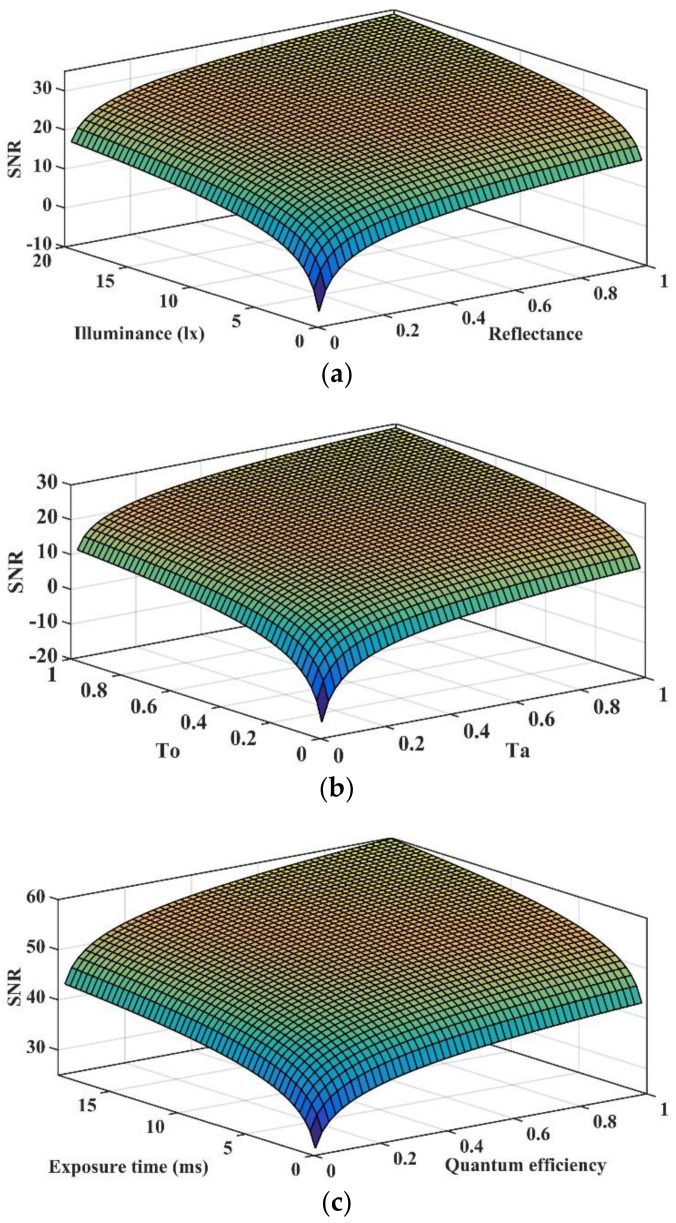
Influences on SNR result from the (**a**) ground object reflectance and illuminance, (**b**) atmospheric transmittance and optical transmittance, and (**c**) quantum efficiency and exposure time.

When the illuminance exceeds this threshold, the growth of SNR slows down and tends to converge gradually with the continuous increase of illuminance. Except the curves of Columbia, the curves of New Delhi and Mexico City shown that the actual on-orbit SNR are much higher than the theoretical SNR. To explain these differences, we analyzed the sensitivity of the SNR model from the aspects of ground objects characteristics, atmospheric characteristics, and detector parameters. Figure 10 illustrates the influences of different factors on SNR.

We can see from Figure 10 that the change of any parameter will greatly influence the values of SNR while the other parameters remain unchanged, however the SNR will converge eventually. The SNR is very sensitive to the atmospheric transmittance. Under bad weather conditions, the SNR will become negative as shown in Figure 10b. In addition, almost all the factors that affect SNR are related to spectral characteristics. Appropriate extension of exposure time is almost the only way to improve on-orbit SNR after the satellite is launched. Now, we can explain the differences between on-orbit SNR and theoretical SNR in Figure 9, which mainly focus on the following aspects:

• Atmospheric factors

In order to obtain clear images, the Luojia 1-01 satellite generally works in good weather, when the atmospheric transmittance is relatively high. The images of selected regions are captured in different time, and the atmospheric condition at that time are different. Thus, there will be difference between average atmospheric transmittance and actual atmospheric transmittance.

• Spectral factors

The atmospheric transmittance, optical transmittance, quantum efficiency, spectrum power function, and the energy of photons are functions of wavelength, related to the spectral characteristics of light sources, thus these corresponding parameters are also different. The theoretical SNR model in this paper considers the central wavelength only, which will lead to errors beyond doubt.

• Target factors

Due to the complex reflection characteristics of ground objects, it is difficult to study them in detail. The typical reflectance of ground objects under night-light is about 0.3. The theoretical SNR model takes this value as standard reflectance to reduce calculation difficulty, which will result in difference between measured data and theoretical data.

The time-sequence-based method makes up for the limitation that the spatial-sequence-based method cannot be utilized for low-resolution cameras, but they have the common calculation steps, thus the former can be regard as a supplement or extension of the latter to some extent. It should be pointed out that in these three cases, the selected regions are large cities, the scattering properties of ground objects are the same or similar in all directions. In other words, our method is limited by the bidirectional distribution characteristics and only suitable for isotropic objects. From the perspective of automation, these two kinds of methods require us to select sampling regions manually, meanwhile the accuracy will be affected by the error caused by human operation. Judging from the calculation speed, the new method is more time-consuming than variance method. However, due to the lack of radiometric calibration data and original images of other night-light remote sensing satellites, the study on the robustness of this proposed time-sequence-based method is insufficient, we would like to use more satellite data to test this method in the future if possible.

## 6. Conclusions

In this paper, considering the characteristics that the illuminance of ground objects is low and varies dramatically under night-light, we propose an on-orbit signal-to-noise ratio (SNR) test method based on time-sequence images for low-resolution night-light cameras. Different from the traditional methods that are based on spatial-sequence images, this new method breaks through the limitation that the traditional methods cannot be applied to low-resolution night-light cameras due to the inability to extract a large area with uniform brightness. We established the radiative transfer model between night-light camera and ground objects. Then we established a theoretical SNR model suitable for night-light cameras based on the analysis of signal electrons and noise electrons. Taking the Luojia 1-01 satellite as reference, and combing with radiometric calibration results, we calculated the theoretical SNR and the actual on-orbit SNR using the camera’s parameters and a series of time-sequence images captured by the Luojia 1-01 satellite. We can conclude from the results that:

(1) By extracting the data of the same object from a series of time-sequence images that were captured during consecutive time, the new method solved the problem that the traditional method cannot be used for low-resolution images, which provides a fast, simple, and reliable solution for on-orbit SNR calculation of low-resolution night-light cameras.

(2) For the Luojia 1-01 satellite, when the image detector works in HDR high-gain mode, the on-orbit test results and the theoretical prediction results have the same variation trend and data distribution properties, all the results are higher than the requirement of applications, which ensures the detection and recognition capability of night-light camera.

(3) According to these three cases, the new method is limited by the bidirectional distribution characteristics and suitable for ground objects with isotropic properties such as cities. For object with anisotropic scattering, using this method may result in large errors and low accuracy.

(4) According to the sensitivity analysis, those parameters related to the spectrum characteristics are the main factors that influence the SNR results, in which atmospheric transmittance is the most sensitive factor. Reasonable setting of camera parameters can effectively improve SNR. The sensitivity analysis also explained the differences between results of actual theoretical SNR and on-orbit SNR. Those spectral-related parameters are too complex to acquire so that they are replaced by the average values in operation waveband. With respect to the results, this kind of simplify is reasonable and the errors are acceptable for night-light image application.

## Figures and Tables

**Table 1:** Spatial resolution of existing night-light remote sensing satellites.

Satellite	Satellite Payload	Spatial Resolution
DMSP	OLS	2700 m @ 850 km ^1^
Suomi NPP	VIIRS	740 m @ 830 km
SAC-C	HSTC	200~300 m @ 705 km
SAC-D	HSC	200 m @ 661 km
Luojia 1-01	-	129 m @ 645 km
International Space station	-	30~50 m @ 300~450 km
Jilin-1 Smart Verification Satellite	-	5 m @ 638 km
EROS-B	PIC-2	0.7 m @ 520 km

^1^ The symbol “2700 m @ 850 km” here means “at the height of 850 km, the spatial resolution is about 2700 m”. And the same symbol also appeared in the latter tables.

**Table 2:** Parameters of the Luojia 1-01 satellite.

Parameters	Symbol	Values
Operation waveband	*λ*	0.5~0.9 μm @ 0.625 μm
Optical transmittance	*t_o_*	70%
GSD	GSD	129 m @ 645 km
Relative aperture	1/F	1:2.8
Central obscuration	*ε*	0

**Table 3:** Parameters of the image detector.

Parameters	Symbol	Values
Dark current	*D_e_*	31.28e^−^/s/pixel @ 25 °C
Readout noise	*σ_read_*	1.47e^−^
Full well capacity	*N_FW_*	120ke^−^
Pixel size	*A_d_*	11 × 11 μm^2^
Integral series	*M*	1
Quantization bits	*b*	15 bits

**Table 4:** Working status and parameter setting of image detector.

Region	Exposure Time	Logging Mode	Gain	Bit Depth	Image Number
Mexico City	13.7 ms	HDR	1.85x	16 bits	13
New Delhi	13.7 ms	HDR	3.68x	16 bits	10
Columbia	13.7 ms	HDR	1.85x	16 bits	19

**Table 5:** Radiometric calibration efficiency under various detector settings.

Gain	Exposure Time	HDR Low-Gain Mode		HDR High-Gain Mode	
		Slope	Intercept	Slope	Intercept
1.85x	2 ms	2263.10	177.71	17,025.89	219.97
	5 ms	4566.74	191.77	40,291.63	186.73
	10 ms	8903.24	196.49	84,850.39	167.80
	18.8 ms	16,253.92	227.31	157,173.66	166.79
3.68x	2 ms	3932.83	201.42	36,073.88	197.02
	5 ms	8797.50	189.43	85,919.26	179.34
	10 ms	17,092.27	225.21	171,150.67	172.92
	18.8 ms	32,913.00	204.48	337,970.41	113.84

**Table 6:** Statistics data of the on-orbit SNR test.

Region	Sampling	Output	SNR	Radiance	Illuminance
Mexico City	13 × 9	208~2557	20.03~42.94	3.10 × 10^−4^~2.06 × 10^−2^	1.62~107.55
New Delhi	10 × 11	193~1326	15.84~39.93	4.94 × 10^−4^~5.41 × 10^−3^	2.58~28.27
Columbia	19 × 6	266~1560	22.10~33.01	1.17 × 10^−3^~1.16 × 10^−2^	6.12~60.70

## References

[1] Hu K., Qi K., Guan Q., Wu C., Yu J., Qing Y., Zheng J., Wu H., Li X. (2017). A Scientometric Visualization Analysis for Night-Time Light Remote Sensing Research from 1991 to 2016. Remote Sens..

[2] Elvidge C.D., Pettit D.R., Cinzano P., Sutton P.C., Small C. Overview of the Nightsat mission concept. Proceedings of the 2007 Urban Remote Sensing Joint Event.

[3] Baugh K., Elvidge C.D., Ghosh T., Ziskin D. (2013). Development of a 2009 Stable Lights Product using DMSP-OLS data. Proc. Asia Pac. Adv. Netw..

[4] Huang Q., Yang X., Gao B., Yang Y., Zhao Y. (2014). Application of DMSP/OLS Nighttime Light Images: A Meta-Analysis and a Systematic Literature Review. Remote Sens..

[5] Chen Y.L., Chen Y.H., Chen C.J., Wu B.F. Nighttime Vehicle Detection for Driver Assistance and Autonomous Vehicles. Proceedings of the International Conference on Pattern Recognition.

[6] Elvidge C.D., Safran J., Tuttle B., Sutton P., Cinzano P., Pettit D., Arvesen J., Small C. (2007). Potential for global mapping of development via a nightsat mission. Geojournal.

[7] Wu R., Yang D., Dong J., Zhang L., Xia F. (2018). Regional Inequality in China Based on NPP-VIIRS Night-Time Light Imagery. Remote Sens..

[8] Zhang Q., Seto K. (2013). Can Night-Time Light Data Identify Typologies of Urbanization? A Global Assessment of Successes and Failures. Remote Sens..

[9] Gao B., Huang Q., He C., Ma Q. (2015). Dynamics of Urbanization Levels in China from 1992 to 2012: Perspective from DMSP/OLS Nighttime Light Data. Remote Sens..

[10] Liu Z., He C., Zhang Q., Huang Q., Yang Y. (2012). Extracting the dynamics of urban expansion in China using DMSP-OLS nighttime light data from 1992 to 2008. Landsc. Urban Plan.

[11] Zhang Q., Seto K.C. (2011). Mapping urbanization dynamics at regional and global scales using multi-temporal DMSP/OLS nighttime light data. Remote Sens. Environ..

[12] Shi K., Yu B., Huang Y., Hu Y., Yin B., Chen Z., Chen L., Wu J. (2014). Evaluating the Ability of NPP-VIIRS Nighttime Light Data to Estimate the Gross Domestic Product and the Electric Power Consumption of China at Multiple Scales: A Comparison with DMSP-OLS Data. Remote Sens..

[13] Tamir R., Lerner A., Haspel C., Dubinsky Z., Iluz D. (2017). The spectral and spatial distribution of light pollution in the waters of the northern Gulf of Aqaba (Eilat). Sci. Rep. UK.

[14] Spitschan M., Aguirre G.K., Brainard D.H., Sweeney A.M. (2016). Variation of outdoor illumination as a function of solar elevation and light pollution. Sci. Rep. UK.

[15] Jiang W., He G., Long T., Guo H., Yin R., Leng W., Liu H., Wang G. (2018). Potentiality of Using Luojia 1-01 Nighttime Light Imagery to Investigate Artificial Light Pollution. Sensors.

[16] Hölker F., Moss T., Griefahn B., Kloas W., Voigt C.C., Henckel D., Hänel A., Kappeler P.M., Völker S., Schwope A. (2010). The Dark Side of Light: A Transdisciplinary Research Agenda for Light Pollution Policy. Ecol. Soc..

[17] Chen Z., Jiang W., Wang W., Deng Y., He B., Jia K. (2018). The Impact of Precipitation Deficit and Urbanization on Variations in Water Storage in the Beijing-Tianjin-Hebei Urban Agglomeration. Remote Sens..

[18] Yang W., Luan Y., Liu X., Yu X., Miao L., Cui X. (2017). A new global anthropogenic heat estimation based on high-resolution nighttime light data. Sci. Data.

[19] Meng C., Dou Y. (2016). Quantifying the Anthropogenic Footprint in Eastern China. Sci. Rep. UK.

[20] Du M., Wang L., Zou S., Shi C. (2018). Modeling the Census Tract Level Housing Vacancy Rate with the Jilin1-03 Satellite and Other Geospatial Data. Remote Sens..

[21] Liu Q., Sutton P.C., Elvidge C.D. (2011). Relationships between Nighttime Imagery and Population Density for Hong Kong. Proc. Asia-Pac. Adv. Netw..

[22] Bharti N., Tatem A.J. (2018). Fluctuations in anthropogenic nighttime lights from satellite imagery for five cities in Niger and Nigeria. Sci. Data.

[23] Li X., Liu S., Jendryke M., Li D., Wu C. (2018). Night-Time Light Dynamics during the Iraqi Civil War. Remote Sens..

[24] Elvidge C.D., Baugh K.E., Dietz J.B., Bland T., Sutton P.C., Kroehl H.W. (1999). Radiance Calibration of DMSP-OLS Low-Light Imaging Data of Human Settlements. Remote Sens. Environ..

[25] Zhang G., Li L., Jiang Y., Shen X., Li D. (2018). On-Orbit Relative Radiometric Calibration of the Night-Time Sensor of the LuoJia1-01 Satellite. Sensors.

[26] Zhu B., Wang X.H., Tang L.L., Li C.R. (2010). Review on Methods for SNR Estimation of Optical Remote Sensing Imagery. Remote Sens. Technol. Appl..

[27] Su Z., Zhong X., Zhang G., Li Y., He X., Wang Q., Wei Z., He C., Li D. (2019). High Sensitive Night-time Light Imaging Camera Design and In-orbit Test of Luojia1-01 Satellite. Sensors.

[28] Cao Y.M., Zhang W., Cong M.Y. (2007). Analysis of Signal-to-Noise Ratio Calculation for Satellite-Based Infrared Staring Sensor. J. Astronaut..

[29] Dietrich O., Raya J.G., Reeder S.B., Reiser M.F., Schoenberg S.O. (2007). Measurement of signal-to-noise ratios in MR images: Influence of multichannel coils, parallel imaging, and reconstruction filters. J. Magn. Reson. Imaging.

[30] Li L., Li M., Zhang Z., Huang Z.L. (2016). Assessing low-light cameras with photon transfer curve method. J. Innov. Opt. Heal. Sci..

[31] Reeder S.B., Wintersperger B.J., Dietrich O., Lanz T., Greiser A., Reiser M.F., Glazer G.M., Schoenberg S.O. (2005). Practical approaches to the evaluation of signal-to-noise ratio performance with parallel imaging: Application with cardiac imaging and a 32-channel cardiac coil. Magn. Reson. Med..

[32] Liu J.G., Zhang L.F., Tong Q.X. (1999). Estimation of Signal to Noise Ratio of Remote Sensing Images. J. Basic Sci. Eng..

[33] Roger R.E., Arnold J.F. (1996). Reliably estimating the noise in AVIRIS hyperspectral images. Int. J. Remote Sens..

[34] Li H.Z., Tian Y., Han C.Y., Wu G.D., Ma D.M. (2006). Assessment of signal-to-noise ratio of space optical remote sensor using artificial neural network. Opto-Electron. Eng..

[35] Zheng X.F., Lin Z.J., Fan L., Guo Y., Xu H.F., Liu Y.G. (2008). Research on Methods for Computing the Volume of Information of Remote Sensing Image. J. Shandong Univ. Sci. Technol. (Nat. Sci.).

[36] Hillger D.W., Haar T.H.V. (2009). Estimating noise levels of remotely sensed measurements from satellites using spatial structure analysis. J. Atmos. Ocean. Technol..

[37] Gao B.C. (1993). An operational method for estimating signal to noise ratios from data acquired with imaging spectrometers ☆. Remote Sens. Environ..

[38] Gao L.R., Zhang B., Zhang X., Shen Q. (2007). Study on the Method for Estimating the Noise in Remote Sensing Images on Local Standard Deviation. J. Remote Sens..

[39] Li X., Li D., Ma R., Zhang Q., Liu S., He T., Zhao L. (2019). Anisotropic characteristic of artificial light at night-Systematic investigation with VIIRS DNB multi-temporal observations. Remote Sens Environ..

[40] Smith W.J. (2007). Modern Optical Engineering.

[41] Thomas G.E., Stamnes K. (2000). Radiative Transfer in the Atmosphere and Ocean. Phys. Today.

[42] Wan Z., Ren J.W., Li X.S., Zhao G.J., Ren J.Y. (2008). Analysis of signal-to-noise ratio for remote sensing TDI CCD camera based on radiative transfer model. Infrared Laser Eng..

[43] Bazell R.J. (1971). Star Bright, Street Light, Which Will They See Tonight?. Science.

[44] Coesfeld J., Anderson S., Baugh K., Elvidge C., Schernthanner H., Kyba C. (2018). Variation of Individual Location Radiance in VIIRS DNB Monthly Composite Images. Remote Sens..

[45] Elvidge C.D., Keith D.M., Tuttle B.T., Baugh K.E. (2010). Spectral identification of lighting type and character. Sensors.

[46] Yu D.Y., Tan H.Y. (2006). Engineering Optics.

[47] Qi K., Hu Y.N., Cheng C., Chen B. (2017). Transferability of Economy Estimation Based on DMSP/OLS Night-Time Light. Remote Sens..

[48] Mao K.B., Qin Z.H. (2004). The Transmission Model of Atmospheric Radiation and the Computation of Transmittance of MODTRAN. Geomat. Spat. Inform. Technol..

[49] Hide R. (1976). Optics of the Atmosphere: Scattering by Molecules and Particles.

[50] Fan X., Zheng W., Singh D.J. (2014). Light scattering and surface plasmons on small spherical particles. Light Sci. Appl..

[51] O’Neil S. (2005). Accurate Atmospheric Scattering. GPU Gems.

[52] Bruneton E., Neyret F. (2008). Precomputed Atmospheric Scattering. Comput. Graph. Forum.

[53] Janesick J.R. (2007). Photon Transfer.

[54] Jacquot B.C., Bolla B.M., Maguire S. Hybrid approach to mean-variance and photon transfer measurement. Proceedings of the Image Sensing Technologies: Materials, Devices, Systems, & Applications II.

[55] Liu Z.X., Wan Z., Li S.X., Li B.Y., Shao Y.R. (2015). Influence factors on SNR of TDICCD space camera. Opt. Precis. Eng..

[56] Zhang C., Sun S.L., Shi W.X., Wang F., Deng D.X. (2016). Linear CCD camera System for industry measurement and its noise evaluation. Opt. Precis. Eng..

[57] Hardis J.E. 100 years of photometry and radiometry. Proceedings of the SPIE—The International Society for Optical Engineering.

[58] Simonot L., Boulenguez P. (2013). Generalization of the geometric description of a light beam in radiometry and photometry. J. Opt. Soc. Am. A Opt. Image Sci. Vis..

[59] Pulli T., Dönsberg T., Poikonen T., Manoocheri F., Kärhä P., Ikonen E. (2015). Advantages of white LED lamps and new detector technology in photometry. Light Sci. Appl..

